# Red Blood Cell Morphology Is Associated with Altered Hemorheological Properties and Fatigue in Patients with Long COVID

**DOI:** 10.3390/biology13110948

**Published:** 2024-11-19

**Authors:** Marijke Grau, Alena Presche, Anna-Lena Krüger, Wilhelm Bloch, Björn Haiduk

**Affiliations:** 1 Molecular and Cellular Sports Medicine, Institute of Cardiovascular Research and Sports Medicine, German Sport University Cologne, 50933 Cologne, Germany; 2 S.P.O.R.T. Institut, Institute of Applied Sports Sciences, 51491 Overath, Germany; haiduk@sportinstitut.net

**Keywords:** Long-COVID, red blood cells, mechanical sensitivity index, morphology, RBC deformability, RBC aggregation, fatigue

## Abstract

Background: SARS-CoV-2 infection adversely affects rheological parameters, particularly red blood cell (RBC) aggregation and deformability, but whether these changes persist in patients suffering from Long-COVID (LC) and whether these changes are related to RBC morphology remain unknown. Methods: Venous blood was collected from *n* = 30 diagnosed LC patients and *n* = 30 non-LC controls and RBC deformability, RBC aggregation, and hematological parameters were measured. In addition, RBCs were examined microscopically for morphological abnormalities. The mechanical sensitivity index (MS) was assessed in *n* = 15 LC and *n* = 15 non-LC samples. Results: Hematological parameters did not differ between the groups. However, LC showed higher aggregation-related parameters. Although RBC deformability was higher in LC, MS, reflecting the functional capacity to deform, was limited in this group. RBCs from LC showed significantly more morphological abnormalities. The extent of morphological abnormalities correlated with MS and the FACIT-Fatigue score of the LC patients. Conclusion: RBCs from LC show a high degree of morphological abnormalities, which might limit the blood flow determining RBC properties and also be related to fatigue symptomatology in LC. Approaches are now needed to understand the underlying cause of these alterations and to ameliorate these permanent changes.

## Simple Summary

SARS-CoV-2 alters the properties of oxygen-carrying red blood cells (RBCs) through a possible deterioration of hemorheological properties, such as aggregation and deformability. However, long-term changes in these properties and a possible association with morphological abnormalities remain unknown. Therefore, this study aims to investigate changes in the above-mentioned RBC properties in Long-COVID (LC). Venous blood was collected from *n* = 30 diagnosed LC and *n* = 30 non-Long-COVID controls (non-LC). Hematological parameters were measured, as well as the aggregation, deformability, and morphology of the RBCs and the mechanical sensitivity index (MS), which reflects the functional capacity of RBCs to deform. The results indicate that hematological parameters were not altered in LC. However, LC showed higher overall aggregation parameters. RBC deformability was higher in LC compared to non-LC; however, MS was limited in this group. LC showed a higher percentage of RBCs with abnormal shapes, which was related to MS and to fatigue, which is considered the leading symptom of LC. It is concluded that the symptoms of LC and changes in the blood flow determining the properties of RBCs are related to the morphological changes in RBCs. Future studies should investigate the underlying causes in order to develop appropriate therapies for this relatively new disease.

## 1. Introduction

Severe acute respiratory syndrome coronavirus-2 (SARS-CoV-2) has been identified as the cause of COVID-19 in 2020 with more than 776 million cases reported to the WHO to date [1]. COVID-19 disease can be mild or lead to severe complications including pulmonary disease, neurological symptoms, and cardiovascular disease such as myocarditis, heart failure, or thromboembolism due to increased blood clotting. Muscular symptoms such as muscle pain, reduced mobility, muscle weakness, and reduced performance have also been described [2,3]. Long-term sequelae of COVID-19 were first reported in early June of 2020 [4] and in July 2020, Carfi and colleagues revealed that over 87% of COVID-19 patients report long-term symptoms [5]. From then on, the number of persons suffering from continuing symptoms after SARS-CoV-2 infection increased. Long-COVID refers to health complaints after a SARS-CoV-2 infection that last beyond the acute phase of the disease of 4 weeks. These symptoms might persist, recur, or reappear after the infection. Post-COVID refers to new or recurrent symptoms after 12 weeks of SARS-CoV-2 infection [3,6]. Thus, the term Long-COVID also includes post-COVID and will be used hereafter.

Long-COVID is considered a global burden affecting both hospitalized and non-hospitalized COVID-19 patients. Studies suggest that 6–15% of COVID-19 patients might have long-lasting symptoms [7,8,9]. A recent study indicates that the prevalence of Long-COVID remains high, despite a high vaccination rate and the availability of antiviral therapies [10]. More than 200 symptoms have been associated with Long-COVID. Lippi et al. summarized that 78% of Long-COVID patients suffer from fatigue. Fatigue describes an extreme form of tiredness and exhaustion related to a disease and its treatment. This extreme form of physical, mental, and emotional exhaustion does not improve with periods of rest. Long-COVID is also associated with a high prevalence for dyspnea (78%), cognitive impairment/brain fog (74%), memory issues (65%), various forms of pain (52–64%), sleep problems (62%), or depression (50%) [11].

The mechanisms responsible for Long-COVID remain unresolved due to the heterogeneity of reported symptoms and the wide variety of individual limitations. Recent reports hypothesize a persistence of SARS-CoV-2 [12] affecting multiple organ systems, including immune dysregulation, microbiota dysbiosis, immune priming, dysfunction of the red blood cell (RBC) system and endothelial abnormalities, dysfunctional neurological signalling [13], and possible skeletal muscle disturbances [14].

Regarding the RBC system, recent evidence suggests that RBCs from Long-COVID patients showed impaired oxygen binding to hemoglobin [15]. Impaired oxygen capacitance and oxygen carrying capacity, as well as a shift in the hemoglobin oxygen dissociation curve, have been reported during COVID-19 disease [16,17] and thus, a permanent alteration of RBCs by the virus seems plausible. In addition, significant changes in the RBC system during COVID-19 include altered cell morphology [18,19], possibly caused by an infection of erythroid progenitor cells, resulting in hematopoietic stress [16,20]. However, alterations in microvascular blood flow determining properties such as RBC deformability, aggregation, and viscosity [17,21,22,23,24,25] have also been reported, as well as limitations in RBC function [17,26]. Thus, SARS-CoV-2-related changes in oxygen delivery might be associated with altered hemorheological properties [17]. Previous studies have suggested that these hemorheological changes persist beyond the acute phase of COVID-19 disease [22]; however, the tested patient cohort did not suffer from Long-COVID. Thus, it remains to be investigated whether hemorheological parameters and RBC aggregation and deformability, in particular, are still impaired in Long-COVID and whether these changes are related to hematological and/or morphological changes. Data are also lacking on a possible relation between symptoms of fatigue and RBC morphological changes in Long-COVID. This is particularly important in view of the fact that changes in the RBCs, in general, and here in particular in Long-COVID, can have an effect on all other body systems and may provide an explanation for the symptoms described. It is hypothesized that the RBCs of patients with Long-COVID are significantly altered, that these changes are associated with altered cell properties, and that an impact of these changes on one of the predominant symptoms of Long-COVID—fatigue—can be demonstrated.

For this purpose, in this cross-sectional study, data from Long-COVID patients were compared with non-Long-COVID controls with respect to RBC-related hematological and hemorheological variables, as well as RBC morphology. In addition, the mechanical sensitivity index (MS), which represents the functional capacity of RBCs to deform [27], was compared between the two tested groups in a sub-experiment. Finally, the morphological changes were related to MS and fatigue symptoms in patients suffering from Long-COVID. The study aimed to expand our knowledge of RBC changes in Long-COVID in order to better understand the disease and identify target points for possible therapies.

## 2. Materials and Methods

### 2.1. Study Cohort

This study was approved by the ethical review board of the German Sport University Cologne (reference number 171/2022). Written informed consent was obtained from all participants. A U09.9 diagnosis (“Post COVID-19 condition, unspecified”) from a medical doctor was required. Information of the study participants are summarized in Table 1. Non-Long-COVID control persons were significantly taller compared to the Long-COVID group, but the remaining parameters were comparable between the tested groups.

Participants with Long-COVID received an average of 3 COVID-19 vaccinations (range 2–5) and the average time between SARS-CoV-2 infection after which the respective Long-COVID-associated symptoms have persisted and blood sampling was 42 ± 26 weeks (range 13–120). Reported Long-COVID-associated symptoms included musculoskeletal symptoms (e.g., muscle pain), cognitive/neurological symptoms (e.g., poor concentration), chest symptoms (e.g., shortness of breath), and anxiety/depression/sleep disturbance. All Long-COVID subjects confirmed that they had symptoms that could be related to fatigue. Therefore, the FACIT-Fatigue scale [28], a 13-item measure of self-reported fatigue and its impact on daily activities, was administrated to assess whether fatigue was a common Long-COVID-associated symptom in the cohort studied. A resultant score <30 indicates a severe form of fatigue. The FACIT-Fatigue score of participants suffering from Long-COVID was 21.35 ± 8.4 (range 7 to 41). Thus, 4 out of 30 participants received a score >30 and 26 out of 30 participants showed a clinically relevant fatigue score.

Non-Long-COVID controls received an average of 3 COVID-19 vaccinations (range 0–5) and reported having 0-2 SARS-CoV-2 infections prior to blood sampling, with at least 12 weeks between the last infection and blood collection. None of the non-Long-COVID control subjects reported symptoms associated with Long-COVID.

All participants were non-smokers.

### 2.2. Blood Sampling

Venous blood was sampled into sodium heparin S-Monovette^®^ or Serum S-Monovette^®^ (Sarstedt AG&Co KG, Nümbrecht, Germany) and either processed within one hour after sampling (heparin blood) or sent to a medical laboratory for serum ferritin analysis (blood serum).

### 2.3. Measurement of Hematological Parameters

A complete blood count was performed on whole blood using a commercially available blood analyzer (Sysmex Digitana KX-21N, Sysmex, Norderstedt, Germany). Reported hematological parameters included red blood cell number (RBC; ×10^6^/μL), white blood cell count (WBC; ×10^3^/μL); platelets (PLTs; ×10^3^/μL); hemoglobin concentration (Hb; g/dL); hematocrit (Hct; %); mean cellular hemoglobin (MCH; pg); mean cellular volume (MCV; fL); mean cellular hemoglobin concentration (MCHC; g/dL); and RBC distribution width (RDW; %). Serum ferritin (μg/L) and fibrinogen were analyzed only in Long-COVID patients to check whether the ferritin and fibrinogen concentrations were within the normal range. Normal range for ferritin: women: 13–300 μg/L; men: 30–300 μg/L; fibrinogen: 170–420 mg/dL.

### 2.4. Measurement of RBC Rheological Parameters of Deformability and Aggregation

RBC deformability and aggregation were measured using the laser-assisted optical rotational red cell analyzer (LORRCA MaxSis, RR Mechatronics, Zwaag, The Netherlands), as previously described [29]. Whole blood was separated by centrifugation (800× *g*, 10 min) and plasma supernatant and sedimented RBCs were stored in separate clean tubes, while the buffy coat containing leukocytes and platelets was removed.

#### 2.4.1. RBC Deformability

Sedimented RBCs were mixed with PVP (Polyvinylpyrrolidone, viscosity: 29.4 cP, RR Mechatronics, Zwaag, The Netherlands) at a ratio of 1:500. The samples were sheared in a Couette system at 9 consecutive shear stresses between 0.3 and 50 Pa. The resulting maximum deformability (EImax) and the shear stress required for one-half of the maximum deformability (SS1/2) were used to calculate the SS1/2:EImax ratio; with lower values representing higher red blood cell deformability [30].

#### 2.4.2. RBC Aggregation

For aggregation measurements, RBCs were mixed with autologous plasma to achieve a final hematocrit of 40%, which was confirmed using a blood analyzer (see Section 2.3). Samples were fully oxygenated for 15 min with a Roller Mixer (Karl Hecht KG, Sondheim vor der Rhön, Germany) prior to measurement. Samples were measured at 37 °C and investigated by syllectometry. The extent of RBC aggregation was expressed as an aggregation index (AI%) and the kinetics of aggregation were represented by aggregation half-time (t1/2, sec). To demonstrate the threshold shear rate that balances RBC aggregation and disaggregation, an iteration procedure was performed to primarily calculate dIsc min. This parameter defines the minimum change in backscatter intensity during the iteration procedure, representing the shear rate at which RBCs begin to disaggregate (y at dIsc min (s^−1^) [29,31].

#### 2.4.3. Measurement of Mechanical Sensitivity Index

The mechanical sensitivity index (MS) was measured in a subset of *n* = 15 LC and *n* = 15 non-LC controls according to the protocol of Horobin et al. [27]. Whole blood was diluted 1:200 in PVP (viscosity 28.7 cP). A total of 22 measurements per patient were performed using the LORRCA MaxSis to determine the MS. First, baseline deformability was measured. Five shear stress conditions (5 Pa, 25 Pa, 50 Pa, and 75 Pa) were then tested with shear durations of 1 s, 2 s, 4 s, 8 s, and 16 s. After each shear condition, RBC deformability was measured again, as described in Section 2.4.1. Finally, a second baseline deformability measurement was carried out to rule out any influence of the time sequence on the results. Again, the SS1/2:EImax ratio was calculated from each sample. The following equation was used to calculate the mechanical sensitivity index (MS) [27]:

$$MS\left(\%\right)=\frac{SS1/2:{EImax\;ratio}_{n}-SS1/2:{EImax\;ratio}_{con}}{SS1/2:{EImax\;ratio}_{n}}\times 100$$

n: specific duration and magnitude of shear

con: unsheared first baseline measurement

An increased MS thus indicates an impaired functional capacity of RBCs to deform.

### 2.5. Morphological Analyses

For a better illustration, the diluted RBCs from Section 2.4.2 were separated on slides and heat fixed. Pappenheim staining (May Grünwald & GIEMSA; Morphisto GmbH, Offenbach am Main, Germany) was conducted according to the manufacturer’s instructions. Images of the stained RBCs were taken using a Zeiss microscope coupled to a CCD camera (DXC-1850P, Sony, Berlin, Germany). The magnification of the images was 400-fold. Modified from a previously published protocol [18], a total of ten images were taken from each slide, and 200 RBCs were counted. From these RBCs, morphological abnormalities of the categories echinocyte/acantocyte, elliptocyte, dacrocyte, and attached RBCs were counted and the resulting numbers were put in proportion to the total RBC number. Results are presented as a percentage of total RBC count.

### 2.6. Statistics

Statistical data analysis was performed using GraphPad Prism Version 9.5.1 (GraphPad Software, Boston, MA, USA). A significance level of α < 0.05 was set for all statistical tests. Normal distribution was confirmed using the Kolmogorov–Smirnov Test. A one-tailed unpaired *t*-test was performed for the metric variables as part of the mean comparison between LC patients and the non-LC control group. The Pearson correlation coefficient was used to calculate the correlations between RBC morphology and both the FACIT-Fatigue score and mechanical sensitivity index. The Pearson correlation coefficient was also used to calculate correlations between plasma fibrinogen concentration and aggregation parameters. Data are presented as mean values ± standard deviation unless otherwise stated.

## 3. Results

### 3.1. Hematological Parameters

The statistical analysis revealed no difference in the tested hematological parameters between non-Long-COVID controls and participants with Long-COVID (Table 2). Serum ferritin levels were in the normal range in all but two Long-COVID patients. Serum fibrinogen was in the normal range in all but one Long-COVID patient.

### 3.2. RBC Aggregation and Deformability

**Figure 1:**
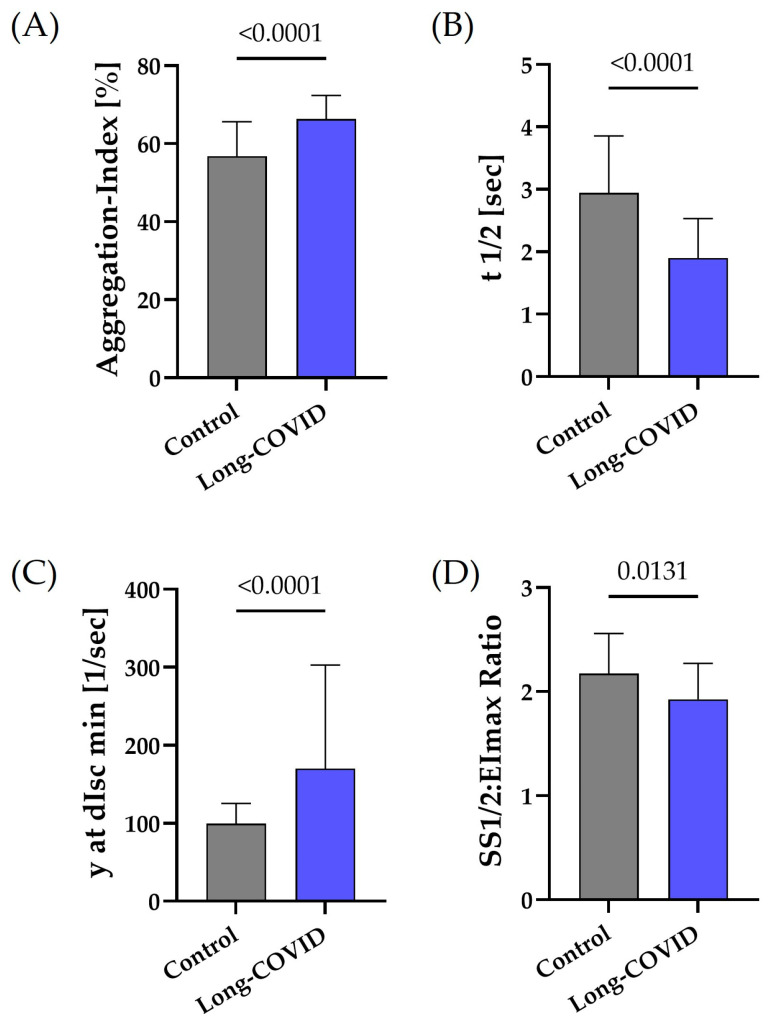
RBC rheological parameters of aggregation and deformability of persons with Long-COVID and non-Long-COVID controls. (**A**) The RBC aggregation index, which reflects the extent of aggregation, was significantly higher in the Long-COVID group than in controls (*p* < 0.0001). (**B**) t1/2, reflecting the speed at which aggregates are formed, was significantly lower in Long-COVID compared to controls (*p* < 0.0001). (**C**) y at dIsc min and the shear rate at dIsc min; the lowest backscatter intensity found during the iteration procedure reflecting the minimum shear rate where RBC aggregates begin to disaggregate was significantly higher in Long-COVID than in controls (*p* < 0.0001). (**D**) The SS1/2:EImax ratio was significantly lower in Long-COVID, reflecting higher deformability (*p* = 0.0131). Data are mean + SD of *n* = 30 per group.

The RBC aggregation index was significantly higher (*p* < 0.0001), the rate at which aggregates form (t1/2) was significantly faster (*p* < 0.0001), and the minimum shear rate at which RBC aggregates begin to disaggregate (y at dIsc min) was significantly higher in Long-COVID (*p* < 0.0001) (Figure 1A–C), thus reflecting higher aggregation compared to non-Long-COVID controls. The SS1/2: EImax ratio was significantly lower in Long-COVID (*p* < 0.05), reflecting higher deformability (Figure 1D). The correlation analysis between fibrinogen concentration and y at dIsc min in Long-COVID patients revealed a low positive correlation (r = 0.3408; *p* = 0.05) and a moderate effect between fibrinogen and AI% (r = 0.4691; *p* = 0.0120).

### 3.3. Mechanical Sensitivity Index

**Figure 2:**
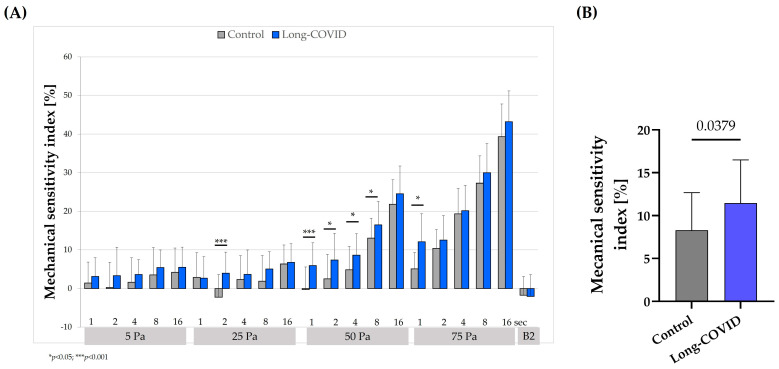
The mechanical sensitivity index of RBC of Long-COVID and non-Long-COVID controls. (**A**) Mechanical sensitivity index was measured for 1, 2, 4, 8, and 16 s at shear stresses of 5, 25, 50, and 75 Pa, respectively. A comparison of the two groups revealed a higher mechanical sensibility index, reflecting impaired mechanical sensitivity in RBCs of Long-COVID for 2 s at 25 Pa, 1, 2, 4, and 8 s at 50 Pa, and 1 s at 75 Pa. (**B**) Averaging all values over time and shear stress revealed a significantly higher mechanical sensitivity index for RBCs from Long-COVID compared to controls (*p* < 0.05). Data are mean + SD of *n* = 15 each. * *p* < 0.05; *** *p* < 0.001.

The mechanical sensitivity index was significantly higher in Long-COVID compared to non-Long-COVID controls for the following shear stresses and durations: 25 Pa and 2 s (*p* < 0.001), 50 Pa and 1 s (*p* < 0.001), 2 s (*p* < 0.05), 4 s (*p* < 0.05), and 16 s (*p* < 0.05), respectively, and 75 Pa and 1 s (*p* < 0.01) (Figure 2A). The calculation of an overall mechanical sensitivity index from all shear stresses and times tested indicated a significantly higher mechanical sensitivity index for Long-COVID compared to non-Long-COVID controls (*p* < 0.05) (Figure 2B). The correlation analysis showed a positive correlation of the mechanical sensitivity index and the percentage of morphologically abnormal RBCs. Pearson’s r was calculated at 5 Pa and 1 s, 25 Pa and 2 s, 50 Pa and 1 s, as well as 75 Pa and 1 s, and the resulting correlation coefficients were 0.2174 (*p* = 0.1286); 0.4067 (*p* = 0.0129); 0.4779 (*p* = 0.0038); and 0.5586 (*p* = 0.0007), respectively, reflecting a moderate correlation for 25 Pa/2 s, 50 Pa/1 s, and 75 Pa/1 s (Figure S1A–D).

### 3.4. RBC Morphology

**Figure 3:**
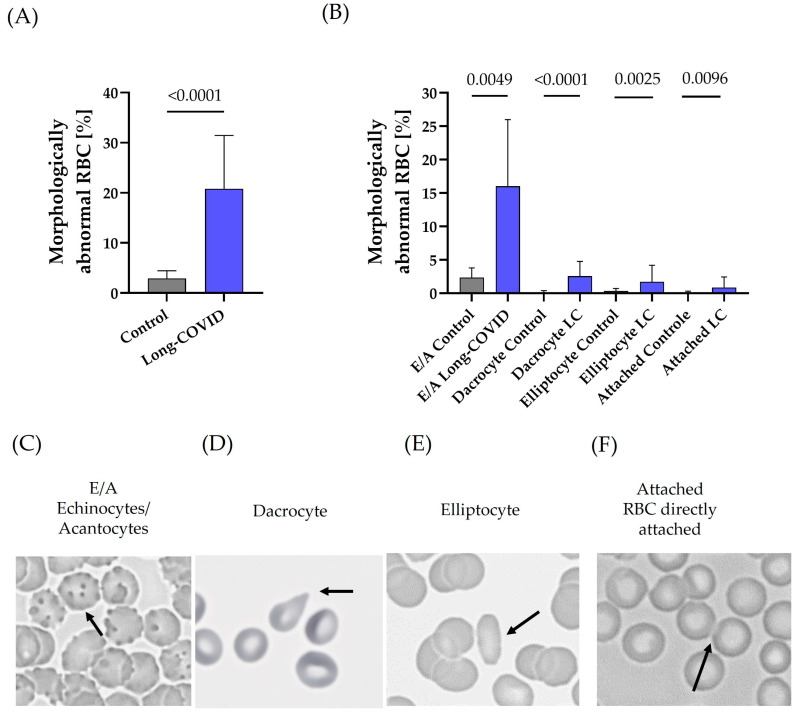
RBC morphology of Long-COVID subjects compared to non-Long-COVID controls. (**A**) A total of 20.8 ± 10.7% of the RBCs from Long-COVID patients showed altered morphology, whereas only 2.9 ± 1.5% of RBCs from the non-LC control group showed altered morphology (*p* < 0.0001). (**B**) Morphologically abnormal RBCs were classified into echinocytes/acantocytes, dacrocytes, elliptocytes, and directly attached RBCs. Individuals with Long-COVID showed a higher percentage in all these categories compared to non-LC controls. Echinocytes/acantocytes accounted for the majority of morphological abnormalities. Data of A + B are mean + SD of *n* = 30 LC and *n* = 30 non-LC controls. Representative images of the categories analyzed, namely (**C**) echinocytes/acantocytes, (**D**) dacrocytes, (**E**) elliptocytes, and (**F**) attached RBCs. Pictures were taken with 400-fold magnification. The arrow points to the corresponding cell showing the respective abnormality.

Participants with Long-COVID showed a significantly higher proportion of morphologically abnormal RBCs compared to non-Long-COVID control subjects, with 20.8 ± 10.7% vs. 2.9 ± 1.5% (*p* < 0.0001) (Figure 3A). These morphological changes were divided into four categories as follows: (a) echinocytes/acantocytes, (b) dacrocytes, (c) elliptocytes, and (d) attached RBCs. Individuals with Long-COVID showed significantly higher proportions in all categories compared to controls (Long-COVID vs. control: (a) *p* < 0.01; (b) *p* < 0.001; (c) *p* < 0.01; (d) *p* < 0.01). Echinocytes/acantocytes represented the category with the highest percentage of RBC abnormalities, with 16.0 ± 9.9% in Long-COVID vs. 2.3 ± 1.4% in controls (Figure 3B). Representative images of the four categories analyzed are shown in (Figure 3C) echinocytes/acantocytes, (Figure 3D) dacrocytes, (Figure 3E) elliptocytes, and (Figure 3F) directly attached RBCs.

### 3.5. Contextual Analyses Between FACIT-Fatigue Score and RBC Shape Changes

The correlation analysis between the FACIT-Fatigue score and the morphological abnormalities of patients suffering from Long-COVID revealed a moderate negative correlation (r = −0.5131; *p* = 0.0061), suggesting that a higher FACIT-Fatigue score is associated with a lower percentage of morphologically abnormal RBCs (Figure S2).

## 4. Discussion

Impairments of the RBC system by SARS-CoV-2 have been well documented in various studies and include, i.e., hemoglobin disturbances [16,21,32], morphological changes [18,21,26], or rheological disturbances [22,24,25], all of which may be related to reduced oxygenation observed in COVID-19 [17]. A variety of symptoms can manifest as a long-term consequence of COVID-19. Yet, it remained unclear whether alterations in RBCs, including changes in shape or hemorheological parameters, are relevant in Long-COVID and might be related to Long-COVID-related fatigue. The main findings of the present study demonstrate a higher percentage of abnormally shaped RBCs in patients suffering from Long-COVID. In parallel, RBC aggregation was significantly higher in this cohort, and although RBC deformability was elevated in Long-COVID, the capacity of RBCs to deform was limited in this study group. A higher percentage of morphological changes correlated in a negative way with the mechanical sensitivity index and in a positive way with fatigue severity. The study parameters were analyzed an average of 42 weeks, or 294 days, after the last SARS-CoV-2 infection. With an average RBC lifespan of 120 days, RBCs would have been replaced twice during this time, ruling out an acute direct effect of the virus on RBCs. This might further indicate that viral RNA/protein persists in the body [12,33,34] and has a lasting effect on the RBC system.

The main function of RBCs is to transport oxygen to the body´s cells and carbon dioxide from the cells. This requires passage through capillaries that are narrower than the diameter of the RBC itself. The ability to adapt the cell shape to these conditions is represented by the cells´ unique deformability. RBC deformability is therefore a key parameter of blood flow in microcirculation. Rigid cells increase flow resistance and decrease tissue perfusion and oxygen delivery [35,36]. Within the microvasculature, RBCs aggregate on the arterial side of the circulatory system and the cells have to disperse to pass through the capillaries. This process can also increase the resistance to flow in microcirculation, and so increased RBC aggregation enhances this effect [37]. Patients with COVID-19 showed higher aggregation and lower RBC deformability, both of which might result in reduced microcirculatory blood flow and oxygen transport to the body cells but might also increase the risk for thrombic events [26,38]. Prolonged decrease in RBC deformability and increased RBC aggregation have been previously described in subjects with initially mild COVID-19 who did not report persistence of COVID-19-associated symptoms and were therefore not considered Long-COVID patients [18,22]. However, these results might suggest that hemorheological parameters might be altered in Long-COVID.

Aggregation parameters analyzed in the present Long-COVID subjects were significantly altered compared to non-Long-COVID subjects, supporting the previous hypothesis. The extent of aggregation (AI%) was significantly higher and t1/2 was significantly lower in Long-COVID. The idea that the formation of large aggregates is associated with faster formation has been described previously [39,40]. The results also suggest a higher minimal shear rate (γ_min_) required to disperse preformed RBC aggregates. Aggregation is influenced by plasma fibrinogen concentration [41], and elevated fibrinogen levels have been associated with hyper-aggregation in Long-COVID patients [25]. Fibrinogen levels measured in the Long-COVID patients in this study were in the normal range, except for one subject, who also had the highest aggregation index with adjusted hematocrit. The correlation analysis revealed a positive correlation between fibrinogen and the aggregation index, indicating that the Long-COVID patients with the lowest fibrinogen levels also had a lower aggregation index. As the resulting correlation coefficient was moderate, further studies with larger sample sizes should extend this finding. Additional cellular factors that may be involved in the alteration of aggregation should also be investigated because the results of Bosek et al. [38] suggest a stronger and more dense aggregate formation in COVID-19, independent of plasma factors such as fibrinogen.

RBC deformability has been shown to equalize with those of healthy control subjects after the acute phase of COVID-19, although a high heterogeneity may remain depending on the cohort studied [22,42]. In contrast, increased RBC deformability after COVID-19 infection in children and adolescents has been reported by Eder and colleagues [43]. The present results from diagnosed Long-COVID patients indicated increased RBC deformability values compared to non-Long-COVID controls, and it might be speculated whether this increase could be considered as a compensatory mechanism counteracting the high aggregation reported here or resulting from damaged cytoskeletal membrane junctions. Lazari et al. [40] have shown that aggregation and deformability might share a common property and further speculated that this mechanism involves the phosphorylation-controlled binding of integral membrane protein complexes containing band 3 and the cytoskeleton. SARS-CoV-2 patients showed fragmentation of cytoskeletal proteins but also of the N-terminal cytosolic domain of band 3, indicating a significant impact of the virus on RBC structure [44]. This might thus explain the significant changes in aggregation and deformability in COVID-19 patients and therefore lead to the speculation that the RBCs of Long-COVID patients could continue to show these changes.

Parameters that affect RBC deformability are cytosolic viscosity, which is influenced by cellular hemoglobin levels, membrane viscoelasticity, and the surface-to-volume ratio, which is reflected by the mean cellular volume [45]. A recent report suggested higher mean cellular hemoglobin and mean cellular hemoglobin concentrations in patients with Long-COVID [15], which could not be confirmed by this study. In the present study, hemoglobin concentration, mean cellular hemoglobin, and mean cellular hemoglobin concentrations of Long-COVID subjects were in the normal range and did not exhibit significant differences compared to the control subjects. Similarly to this dissent, previous reports summarized by Lechuga et al. [46] revealed that, e.g., hemoglobin levels might return back to normal or remain altered in the aftermath of COVID-19. Differences in the outcome might be related to the initial severity of COVID-19 as we have previously shown different hematological outcomes for critically ill COVID-19 patients and those with a mild course of the disease [18,21]. Remaining investigated hematological parameters were comparable between the tested groups, as well as the serum ferritin concentration of the Long-COVID group, which corresponded to normal values, except two of the tested Long-COVID patients, who exhibited serum ferritin values below the normal range, indicating empty iron stores. However, the remaining blood values of these individuals remained unobtrusive. The findings of the present study and the recent suggestions on hematological changes [15,46,47] in Long-COVID illustrates the high individual character of Long-COVID but also suggests that altered hemorheological parameters are not the result of altered hematologic values.

Complementary experiments have been carried out to investigate an additional aspect of RBC deformability, namely the capacity of RBCs to deform, as reflected by the mechanical sensitivity index. An increased mechanical sensitivity, reflecting a reduced functional capacity to deform, may lead to a shift in the hemolytic threshold, which might suggest that those RBCs show a reduced life span. In the present study, the overall MS was significantly higher in patients with Long-COVID, and this was particularly evident at shear stresses > 50 Pa. It could be speculated that this may result in an early hemolysis compared to RBCs from non-Long-COVID subjects. An increase in hemolysis markers in patients with Long-COVID has also been reported by Cervia-Hasler et al. [48]. Although such high-shear-stress conditions are highly unlikely to be present in normal circulation, these values provide information about functional restrictions of RBCs associated with SARS-CoV-2 infection.

In COVID-19 patients, the morphology of RBCs has been shown to be significantly altered [18,26,49], which was also described to affect hemorheological properties [26,50]. To the best of the authors´ knockledge, RBC morphology has not yet been analyzed in patients suffering from Long-COVID. The data presented here therefore show, for the first time, a significantly increased number of morphological changes in the RBCs of Long-COVID patients. Thus, the number of echinocytes/acantocytes were markedly high. In addition, a correlation between morphological changes and MS could be shown, which represents a direct link between the cell shape and cell properties in Long-COVID. The extent of morphological changes also correlates with the fatigue score. This indicates that patients with severe fatigue symptoms also have a higher number of morphologically altered RBCs, suggesting a direct relation between cellular changes and cellular function, making it a leading symptom of Long-COVID.

## 5. Conclusions

This study highlights persistent significant changes in RBC rheological parameters and RBC morphology in Long-COVID patients. These include increased RBC aggregation, altered deformability, and a higher proportion of abnormally shaped RBCs, all of which may collectively negatively affect blood flow and therefore oxygen supply in vivo [51]. The limitation of the study is that the direct effect of the presented RBC changes in blood flow and oxygen availability could not be verified and should thus be investigated in future studies. However, this link seems plausible, as the extent of RBC shape changes correlates with both the capacity of RBCs to deform and fatigue severity. Despite normal hematologic values in the Long-COVID patients, the study suggests persistent cellular changes possibly related to persistent viral effects on RBC structure. Since it was not possible to differentiate the Long-COVID patients according to the severity of the disease, it would be interesting in future studies to classify the Long-COVID patients according to the severity of their symptoms into patients with moderate and severe Long-COVID using the Post COVID-19 Syndrome Score or the Post COVID-19 Functional Status Scale. This would possibly allow us to obtain information whether a correlation can be shown between the overall severity of Long-COVID and the functional RBC parameters described here.

Studies could build on these findings and apply strategies known to improve hemorheological properties, including exercise [52,53], to Long-COVID. This approach might be feasible to improve rheological properties and thus also influence the extent of symptoms of Long-COVID, taking into account the morphology of the RBCs.

## Figures and Tables

**Table 1:** General information of study participants. Data are mean (standard deviation).

	Control	Long-COVID	*p*-Value
Number, n	30	30	
Male/female	18/12	19/11	
Age [years]	55.1 (19.8)	51.3 (14.9)	0.20
Height [m]	1.77 (0.1)	1.72 (0.1)	0.02
Weight [kg]	74.1 (15.1)	76.2 (16.5)	0.42
BMI [kg/m^2^]	23.4 (3.3)	25.7 (5.2)	0.09

**Table 2:** Blood parameters of persons with Long-COVID and non-Long-COVID controls. Data are mean (SD) of *n* = 30 per group.

Parameter	Control	Long-COVID	*p*-Value
WBC (×10^3^/μL)	6.9 (1.9)	6.5 (1.5)	0.17
PLT (×10^3^/μL)	236.1 (63.2)	254.6 (76.1)	0.18
RBC (×10^6^/μL)	4.7 (0.4)	4.9 (0.6)	0.44
Hb (g/dL)	14.2 (1.1)	14.8 (2.0)	0.23
Hct (%)	42.2 (2.9)	43.5 (5.1)	0.28
MCV (fL)	89.3 (3.4)	89.4 (2.9)	0.17
MCH (pg)	30.1 (1.4)	30.4 (1.5)	0.08
MCHC (g/dL)	33.6 (1.1)	33.9 (0.9)	0.28
RDW (%)	12.9 (0.7)	13.1 (0.7)	0.14
Ferritin (μg/L)		63.2 (48.7)	
Fibrinogen (mg/dL)		305.9 (71.2)	

WBC: white blood cell; PLT: platelet; RBC: red blood cell; Hb: hemoglobin concentration; Hct: hematocrit; MCV: mean cellular volume; MCH: mean cellular hemoglobin; MCHC: mean cellular hemoglobin concentration: RDW: RBC distribution width.

## Data Availability

The data that support the findings of this study are available upon reasonable request from the corresponding author. The data are not publicly available due to privacy or ethical restrictions.

## Supplementary Materials

The following supporting information can be downloaded at: https://www.mdpi.com/article/10.3390/biology13110948/s1, Figure S1: Pearson correlation coefficient of mechanical sensitivity index (MS) and percentage of morphologically abnormal RBCs (%); Figure S2: Pearson correlation coefficient of FACIT-Fatigue score and percentage of morphologically abnormal RBCs (%) in patients with Long-COVID.
