# Supplementary material for: Red Blood Cell Morphology Is Associated with Altered Hemorheological Properties and Fatigue in Patients with Long COVID
Source: Biology (Basel). 2024 Nov 19;13(11):948. doi: 10.3390/biology13110948 (PMC11592038; doi:10.3390/biology13110948)
Supplement: Supplementary file 1 [file biology-13-00948-s001.zip › biology-3326596-supplementary.pdf]

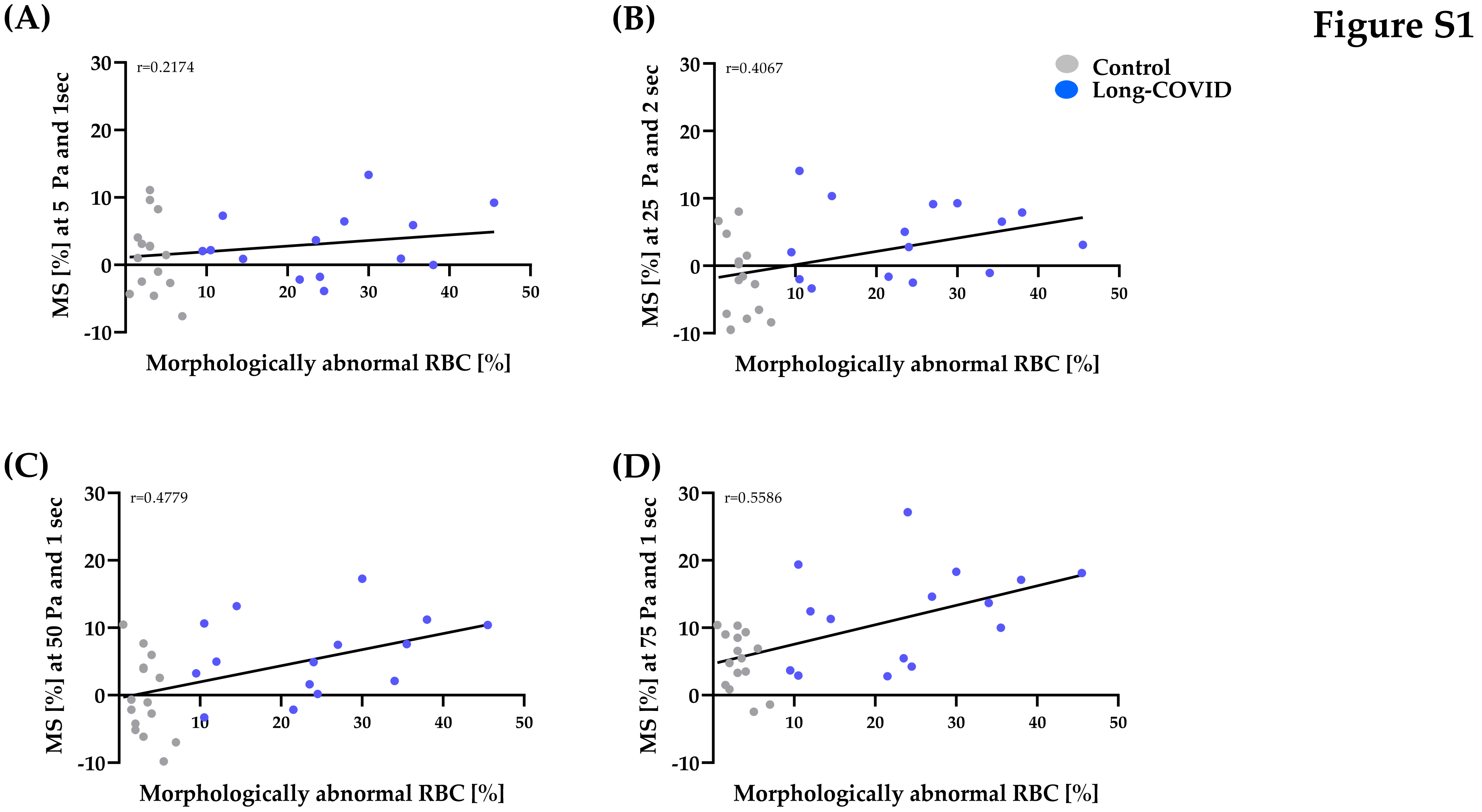

Figure S1: Pearson correlation coefficient of Mechanical sensitivity index (MS) and percentage of morphologically abnormal RBC (%). Pearson´s r was calculated at (A) 5 Pa and 1sec, (B) 25 Pa and 2 sec, (C) 50 Pa and 1 sec and (D) 75 Pa and 1 sec. Resulting correlation coefficient were 0.2174 ($p=0.1286$); 0.4067 ($p=0.0129$); 0.4779 ($p=0.0038$) and 0.5586 ($p=0.0007$), respectively, reflecting a moderate correlation for B-D. Data are n=15 per group. Blue circle: LC; grey circle: non-LC controls.

Figure S2

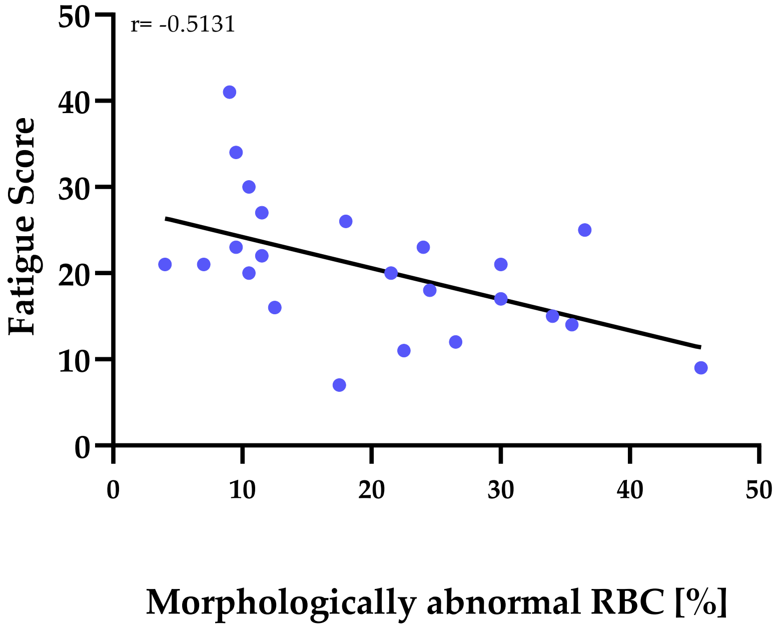

Figure S2: Pearson correlation coefficient of FACIT-Fatigue score and percentage of morphologically abnormal RBC (%) in patients with Long-COVID. Pearson correlation analysis revealed a moderate negative correlation between FACIT Fatigue score and RBC morphology with Pearson r = -0.5131 and p=0.0061. Data are n=30.
